# The Effect of Lightweight Concrete Cores on the Thermal Performance of Vacuum Insulation Panels

**DOI:** 10.3390/ma13112632

**Published:** 2020-06-09

**Authors:** Sang-Yeop Chung, Pawel Sikora, Dietmar Stephan, Mohamed Abd Elrahman

**Affiliations:** 1 Department of Civil and Environmental Engineering, Sejong University, Seoul 05006, Korea; sychung@sejong.ac.kr; 2 Building Materials and Construction Chemistry, Technische Universität Berlin, Gustav-Meyer-Allee 25, 13355 Berlin, Germany; pawel.sikora@zut.edu.pl (P.S.); stephan@tu-berlin.de (D.S.); 3 Faculty of Civil Engineering and Architecture, West Pomeranian University of Technology Szczecin, Al. Piastow 50, 70-311 Szczecin, Poland; 4 Structural Engineering Department, Mansoura University, Elgomhouria St., Mansoura City 35516, Egypt

**Keywords:** lightweight concrete, foam concrete, vacuum insulation panel (VIP), micro-CT, thermal properties

## Abstract

The performance of vacuum insulation panels (VIPs) is strongly affected by several factors, such as panel thickness, design, quality of vacuum, and material type. In particular, the core materials inside VIPs significantly influence their overall performance. Despite their superior insulation performance, VIPs are limited in their widespread use as structural materials, because of their low material strength and the relatively expensive core materials. As an alternative core material that can compensate these limitations, foamed concrete, a type of lightweight concrete with very low density, can be used. In this study, two different types of foamed concrete were used as VIP core materials, with their effects on the thermal behavior of the VIPs having been evaluated using experimental and numerical methods. To confirm and generate numerical models for VIP analysis, micro-computed tomography (micro-CT) was utilized. The obtained results show that insulation effects increase effectively when panels with lightweight concrete are in a vacuum, and both foamed concrete types can be effectively used as VIP core materials.

## 1. Introduction

A vacuum Insulation Panel (VIP) is a system for thermal insulation which consists of an enclosed rigid core and a surrounding membrane. In general, the thermal conductivity of VIPs is about 5 to 10 times lower than other insulation materials or systems [1,2]. There has recently been growing interest in energy efficiency, with the reduction of energy consumption through heat loss having become an important issue in most industries, especially the building sector. To satisfy energy requirements in the construction field, new insulating materials with a high porosity and with reduced material densities have been developed, but further reduction in thermal conductivity is still desirable for more effective insulation [3,4,5]. While VIPs are also being used in buildings for insulation purposes, there are limitations in their widespread application because of the relatively expensive insulation techniques involved, as compared to other insulations [6], such as mineral wool and foamed polystyrene [7,8]. In order to overcome price limitations, a method for reducing VIP production costs is needed.

VIPs are generally composed of two parts: a membrane and a core material. More specifically, VIPs are generally flat and consist of an enclosure or membrane, which are often metalized or laminates made of welded metal and plastic foils, and a core material. The core materials need to be open-porous for effective evacuation, and they need to be capable of withstanding the external load caused by atmospheric pressure and a gas tight envelope, to maintain the required vacuum quality in the production process [9,10]. In addition, they must have long-term stability to be used in buildings. It is well known that relatively expensive materials, such as fumed silica or precipitated silica, are currently used for VIP manufacturing, and these contribute to high costs [11]. Therefore, the use of alternative low cost materials for the VIPs is critical to overcome the overall challenge of high cost.

Concrete and cement-based materials are the most widely used materials in the construction industry, due to advantages, such as low cost, high strength, water resistance, convenient production, as well as multiple applications [12,13]. Compared with the conventional core materials of VIPs, concrete is an economical and versatile material that has advanced mechanical properties, such as compressive strength; the use of concrete for VIPs can therefore reduce production costs. The main role of VIPs is to enhance thermal insulation, with VIPs needing to have low thermal conductivity and a high-porous structure [14]. Among the various cement-based composites, lightweight concrete is a widely used material containing numerous pores within the material and has a density less than 2000 kg/m${}^{3}$, which is lower than that of normal concrete [15,16]. In addition, lightweight concrete has a relatively higher stiffness and strength than conventional VIP core materials.

Lightweight concrete has itself been widely utilized for insulation purposes [17,18]. In general, it is mainly produced by using either lightweight aggregates [19,20,21], or foaming agents [22,23,24], which are called lightweight aggregate concrete and foamed concrete, respectively. Lightweight aggregate concrete contains more than 50 vol.% lightweight aggregates in the concrete volume, while foamed concrete is a cellular material with a highly porous structure. It has been found that lightweight aggregate concrete has a relatively higher strength than foamed concrete, but that foamed concrete demonstrates lower thermal conductivity than lightweight aggregate concrete at the same density level [25]. Foamed concrete is thus more suitable than other lightweight concrete for use as a VIP core since the core material must be a porous media so as to be suitable for evacuation processes. Foamed concrete has very low density ranges, between 200 and 1600 kg/m${}^{3}$, and with a compressive strength of 0.2 to 15 MPa, which is higher than that of the core materials of ordinary VIPs [26,27].

The main purpose of this research was to evaluate the effect of lightweight foamed concrete as a core material on the performance of VIPs. For this purpose, foamed concrete was produced and utilized as the core material for the target VIP. The most important requirements of the core are a porous structure for evacuation, as well as insulation related properties, with the material also needing to have the appropriate strength and stability to withstand the pressure in the vacuum process. Thus, in addition to the pure foamed concrete, another foamed concrete specimen incorporating lightweight aggregate for better strength was also designed and used. VIP specimens with lightweight concrete cores were then manufactured using a membrane and the same procedures as commercial VIP specimens. To confirm the insulation performance of the vacuumed lightweight concrete, the thermal conductivity of the lightweight concrete specimens before and after evacuation was experimentally and numerically measured and compared using standardized measurement devices and finite element analysis, respectively. To visualize and generate the numerical model, micro-computed tomography (micro-CT), a nondestructive method, was used, with the microstructures of the used core materials being subsequently examined [28,29,30]. Following on from the obtained results, the effects of lightweight concrete as a core material are examined and discussed below.

## 2. Sample Preparation

This study focuses on the effects of lightweight concrete on the insulation performance of VIPs, and the appropriate selection of material for the core part of the VIP is important. The core material needs to be porous and have sufficient strength to withstand pressure for effective evacuation. Among several types of lightweight concretes, foamed concrete, which contains numerous pores, was selected. In addition to the pure foamed concrete, another type of foamed concrete containing lightweight aggregates was also prepared and used as a core material. Despite its versatility in insulation, pure foamed concrete with very low density has a relatively low strength and stability in comparison to other types of lightweight concretes including lightweight aggregate concrete. Since conventional lightweight aggregate concrete contains less pores than foamed concrete, it is not an appropriate VIP core material. Alternatively, foamed concrete incorporating lightweight aggregates can be used as the core material, reducing shrinkage and enhancing material strength.

### 2.1. Core Materials

Two different types of lightweight foamed concrete were used in this study: pure foamed concrete and foamed concrete with lightweight aggregates (LWAs). The former specimen was a type of pure foamed concrete, while the latter contained lightweight aggregates (Liaver${}^{®}$) for better strength and less shrinkage. The VIP specimens with pure foamed concrete were denoted as ‘FCP’, while foamed concrete with lightweight aggregates was denoted as ‘FCA’. Two mixes with similar compositions, but with different densities (250 kg/m${}^{3}$ (FCP) and 350 kg/m${}^{3}$ (FCA)), were prepared and examined.

**Figure 1:**
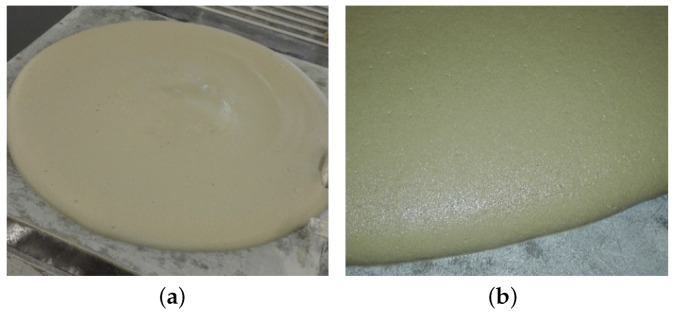
Confirmation of the consistency of the used foamed concrete (FCP): (**a**) general flow test, (**b**) magnification of the edge.

The cement used in this investigation is CEM I 52.5 R conforming EN 197-1 produced by Heidelberg Cement (Heidelberg, Germany). Hard coal fly ash type C (EFA-Füller, Baumineral GmbH, Germany) conforming EN 450-1 was used as a cement replacement (FA); cement was replaced with 30 wt.% of FA. To produce the FCA specimen with lightweight aggregates, fine expanded glass (Liaver${}^{®}$, 0.1–0.3 mm) was added. Chemical and physical characteristics of the used binder are summarized in Table 1. For both mixes, the water-binder ratio (w/b) was fixed at 0.4, while the ratio of foam:paste was fixed at 3:1 and 4:1 to produce materials with two different densities. The used foaming agent was Lightcrete 400 provided by Sika Germany (Stuttgart, Germany). A compatible type of PCE superplasticizer (tailor-made Sika Viscocrete) without destroying the foam bubbles was used to achieve a workable foamed concrete with F3/F4 consistency class in accordance with EN 206-1, as shown in Figure 1. In order to facilitate proper stability and homogeneity of the fresh mix with the foam bubbles, a viscosity-enhancing admixture (stabilizer ST3, Sika, Rosendahl, Germany) was added. Table 2 presents the proportions of the foamed concrete mixes.

To produce the foam, a foam generator SG S9 (Sika GmbH, Rosendahl, Germany) was employed. In the manufacturing process and according to the provider, the foam should be produced continuously to guarantee stable foam bubbles. The applied water and air pressures were adopted at 3 and 2 bars, respectively, with the dosage of foaming agent of 2% to achieve a uniform and steady foam. The density of the foam was measured using 30 L bucket and it ranged between 33–35 g/L. To manufacture foamed concrete, cement slurry was produced then it is mixed with the preformed foam. The absence of coarse aggregates increases the possibility of clumps and agglomeration of fine materials when mixing with water. Therefore, a high-intensity mixer (Eirich Group, Hardheim, Germany) was used to mix the cement paste to ensure homogeneous distribution of particles and avoid agglomeration and clumps of fine materials. After manufacturing the paste, the required foam volume was produced and both were mixed using a concrete mixer (Zyklos, Freisbach, Germany) with 50 L capacity to produce foamed concrete. Fresh properties of concrete were measured; consistency using the flow table was determined by measuring the diameter of the concrete without jolting the table. The density of fresh concrete was measured according to EN 12350-6 using a 5 L bowel but without compaction. Foam bubbles are very light and very sensitive to temperature changes and any movements. Therefore, no compaction or vibration has been applied when filling the 10 × 10 × 10 cm${}^{3}$ molds. The concrete samples were kept at a chamber with controlled humidity and temperature until demolding after 24 h. The curing process was performed at the climate chamber with a temperature of 21 ${}^{{}^{\circ}}$C and relative humidity of 99% until the testing day.

### 2.2. Production of VIPs

**Figure 2:**
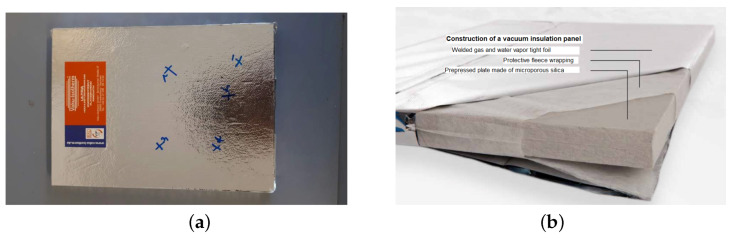
Commercial vacuum insulation panel (VIP) produced by Vaku-Isotherm GmbH (Frankenberg, Germany): (**a**) general product, (**b**) structure of the VIP.

**Figure 3:**
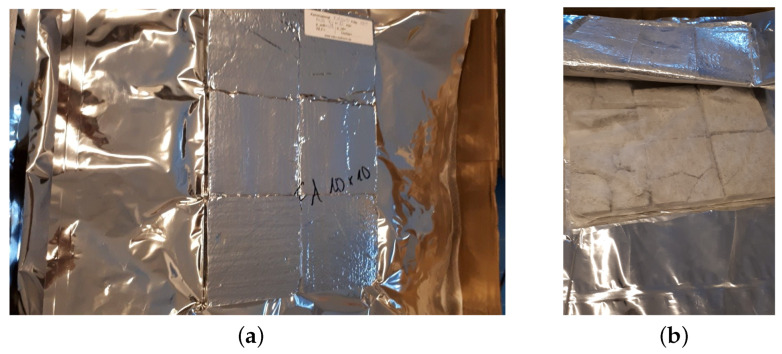
Evacuated foamed concrete (VIP): (**a**) VIP sample (FCP), (**b**) Inside of the VIP sample.

Several methods can be used to evacuate and produce VIP samples, [31,32,33,34]. These studies have confirmed that the evacuation pressure should be less than 0.1 mbar, so as to produce an effective vacuum insulation panel, and that the procedure for producing VIPs with a reliable quality is highly complicated. However, since the main objective of the present study was to investigate the effects of various core materials on VIP properties, the VIP manufacturing process was beyond the scope of the study. The used VIP specimens were produced by Vaku-Isotherm GmbH (Frankenberg, Germany), using developed lightweight foamed concrete samples as the core materials. Figure 2 shows a product made by Vaku-Isotherm GmbH with a core consisting mainly of silica (preferably fumed silica) [32,35]. The VIP specimens with lightweight concrete were manufactured using exactly the same procedure as in Figure 2b, with only the core materials being replaced with lightweight concrete specimens. For measurement, six samples from each mix with dimensions of 10 × 10 × 2 cm${}^{3}$ were prepared and tested, as shown in Figure 3. In this figure, the target VIP sample (Figure 3a) and its internal features (Figure 3b) are presented. To produce a more effective VIP sample, the foamed concrete specimen was covered with double-layered felt and sealed with a metalized plastic film. The obtained VIP system was almost identical to the commercial product in Figure 2.

## 3. Evaluation of Thermal Properties and Microstructures

The most important function of VIPs is to enhance the thermal insulation of buildings or structures, with the thermal properties of target materials, such as thermal conductivity, being potential parameters for evaluating VIP performance. In this study, the thermal conductivity of the VIP specimens before and after evacuation was investigated experimentally and numerically. Two standardized experimental methods were used for accurate measurement, while numerical simulation was performed using micro-CT images. The microstructural characteristics of the core materials were also evaluated with the micro-CT data.

### 3.1. Experimental Measurement

For thermal conductivity measurements to compare insulation performance before and after evacuation, two methods were used to enhance accuracy and to confirm the influence of evacuation on the thermal characteristics of lightweight concrete: the Hot Disk device that satisfies ISO 22007-2 and the hot plate method, according to EN 12667. Both devices satisfy the necessary standards and have been widely used to measure the thermal properties of various objects, including construction materials [25,36,37,38].

**Figure 4:**
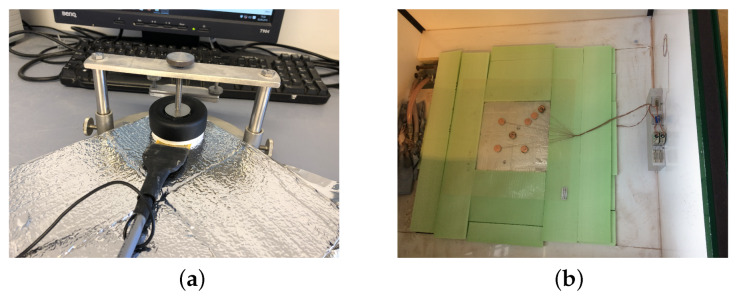
Experimental measurements of thermal conductivity: (**a**) the Hot Disk device, (**b**) the hot plate method.

Figure 4 shows the measurement processes using the Hot Disk (a) and the hot plate (b) methods. From the Hot Disk device, thermal properties, such as specific heat, thermal diffusivity and thermal conductivity, can be effectively and efficiently obtained without complex procedures. For the measurement, a sensor, used as a current supplier and temperature monitor, was positioned between the samples, as shown in Figure 4a; thermal properties can be calculated based on temperature change information [39].

The other method, the hot plate method in Figure 4b, is a standardized approach for measuring the thermal conductivity of target materials. In this method, a sample with a reference material is placed on the top of a calorimetric chamber, with the heat applied from underneath. Based on the known thermal conductivity of the reference material and the heat flux that passes through the two layers, the thermal conductivity of the target material can be ascertained [40,41]. The obtained results using both devices were compared to confirm accuracy and were used for numerical analysis, as input parameters.

### 3.2. Micro-CT Investigation

The lightweight concrete specimens used in this study are highly porous materials, with their pore characteristics strongly affecting their material properties. In particular, the pore structures of VIP core materials are some of the most critical factors in determining VIP performance, and an appropriate analysis is needed. For this purpose, X-ray micro-CT was used here. Micro-CT is a non-destructive method which can be used to visualize the internal microstructure of a target material; both 2D and 3D geometric characteristics can be examined using micro-CT images [28,29,30]. The used micro-CT device is self-made which consists of a Hamamatsu microfocus X-ray source and flat panel detector. The goniometers are from Huber${}^{®}$ (Rimsting, Germany). In detail, the imaging system consists of a closed, air-cooled microfocus X-ray source (spot size 5–50 μm depending on voltage and current) from Hamamatsu${}^{®}$ with up to 150 kV voltage and 500 μA current, corresponding to a power of 75 W, and a flat panel detector is also from Hamamatsu${}^{®}$ [42] (Shizuoka, Japan).

**Figure 5:**
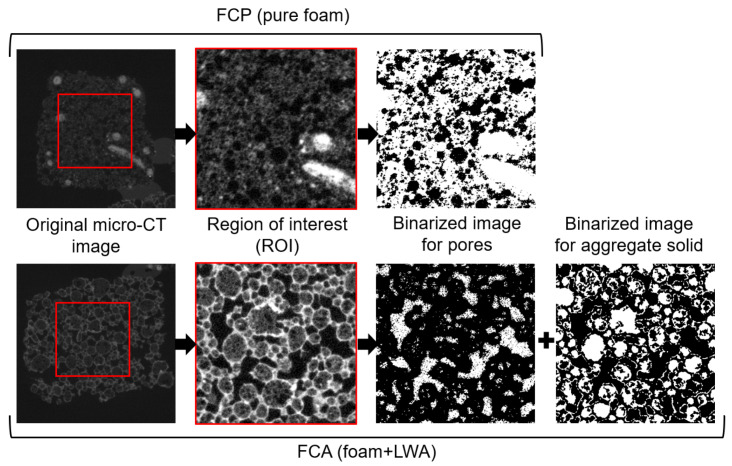
The procedure of micro-CT imaging for the specimens (Note: the 1st figures in both the FCP and FCA specimens are original 8-bit CT images, and the 2nd figures are of the region of interest (ROI) selected from the 1st images. The 3rd images are the segmented images of pores. For the FCP specimen, black represents pores, while white represents the background. In contrast, in the 3rd image of the FCA specimen, the white are pores, and the black is solid including lightweight aggregates. In the 4th FCA image, the white region represents the solid parts of the lightweight aggregates).

**Figure 6:**
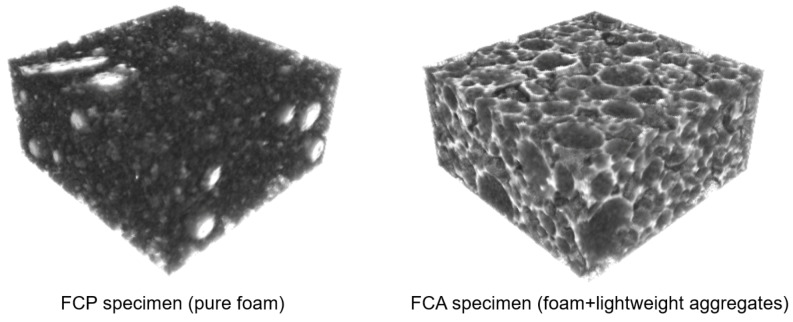
3D micro-CT image of the core materials.

Figure 5 shows an image process for classifying the pore and solid from the FCP and FCA specimens, used for the characterization of the materials. The samples for the micro-CT measurement were selected from the whole specimen which used as a core material of the VIP sample. Cubes of 20 mm edge dimensions were scanned, and a total of 600 slices were recorded. The 1st and 2nd images are the original 8-bit micro-CT image and its region of interest (ROI), respectively. Among the images of the whole specimen, for more effective description, the region of interest (ROI) with 400 × 400 × 200 voxels (*x* × *y* × *z*) was selected, which can be considered as representative volume of the specimens. The ROIs and the binary images in Figure 5 are composed of 400 × 400 pixels with 29.8 μm/pixel. To segment the pores from the ROIs, a threshold was selected using the Otsu method [43] and manual confirmation, with the binary image being generated using the thresholding methods and the imaging toolbox in MATLAB (R2019b) [44]. In the binary image of the FCP specimen, which is pure foamed concrete, the black represents the pores, and the white represents the solid phase. For the FCA specimen, the segmentation is more complex because of the lightweight aggregates in the material. As can be seen in the lower images in Figure 5, lightweight aggregate particles have a more rigid solid than the matrix, which are described in light and dark grayscale according to their relative densities, respectively. This indicates that the aggregate solids are denser than the matrix and have different characteristics and properties. The segmentation of pores, aggregates, and the matrix was conducted using a multi-level thresholding approach [25,45]. As shown in Figure 6, the converted images were subsequently stacked, and the obtained 3D images were utilized for characterization and numerical analysis.

### 3.3. Numerical Analysis

The thermal conductivity of the VIP specimens was also computed using numerical simulation; commercial finite element analysis software, ABAQUS, was used for the analysis [46]. In the heat transfer analysis, the modified governing equation in 3D is described as follows [47]:

(1) $$\frac{\partial T}{\partial t}=\frac{k}{\rho C}\left(\frac{\partial^{2}T}{\partial x^{2}}+\frac{\partial^{2}T}{\partial y^{2}}+\frac{\partial^{2}T}{\partial z^{2}}\right)-\lambda \cdot T^{*}$$

where *T* [K] is the temperature, $T^{*}$ [K] is the surrounding temperature, *t* [s] is the time, *k* [W/m/K] is the thermal conductivity, ρ [kg/m${}^{3}$] is the mass density, and *C* [J/g/K] is the specific heat. In this formulation, heat loss is considered using the heat loss coefficient, λ [1/s].

A weak form of the governing equation can be obtained by integrating Equation (1). Effective heat flux (*q*) is obtained by averaging the heat flux of each element, with the effective thermal conductivity (*k*) then expressed from Fourier’s law, as follows:

(2) k=q·L/△T

where *L* is the characteristics length of the specimen, and △T is the temperature difference in the heat flow direction.

For the thermal analysis, the input bulk parameters (specific heat and thermal conductivity) were obtained experimentally. In particular, the total thermal conductivity of VIPs ($\lambda_{tot}$) is composed of several contributions, as follows [48,49]:

(3) $$\lambda_{tot}=\lambda_{solid}+\lambda_{gas}+\lambda_{rad}$$

in Equation (3), $\lambda_{solid}$ is the solid thermal conductivity, $\lambda_{gas}$ is the gas thermal conductivity, and $\lambda_{rad}$ is the radiation thermal conductivity. Here, $\lambda_{solid}$ is the thermal conductivity of the bulk material, and $\lambda_{gas}$ is the thermal conductivity of air. For a more detailed analysis, $\lambda_{rad}$ also needs to be considered, because radiation effects are found in pores larger than a few micrometers. The value can be calculated using formula [50], with $\lambda_{rad}$ of 0.01 [W/m/K] being used. The used input parameters for the thermal analysis are presented in Table 3. In this table, the density of the aggregates was provided by the manufacturer, and the other properties, such as thermal conductivity and specific heat, were measured using the Hot Disk device. In the case of evacuation, the thermal conductivity of air was set at zero. For boundary conditions, a constant temperature applied to the top surface and a surrounding temperature were set to be 60 ${}^{{}^{\circ}}$C and 26 ${}^{{}^{\circ}}$C, respectively. The bottom surface was considered to have heat loss with a heat loss coefficient of 1.7 [1/s], while the other remaining surfaces were considered to have no heat loss.

## 4. Results and Discussion

The performance of the VIPs with lightweight concrete cores were investigated using the thermal conductivity of the specimens. Both experiments and simulations were conducted to evaluate the thermal conductivity of the specimens, with the effects of the core materials being examined based on their thermal properties.

### 4.1. Experimental Results

The measured VIP thermal conductivity values are presented in Table 4 and Table 5. Table 4 presents the results from the Hot Disk device, while Table 5 shows the result from the hot plate method. All measurements were performed three to five times for each specimen to enhance accuracy, with only the mean values presented here. In the Hot Disk method, three different points were selected, with the thermal conductivity being measured at certain points for a more accurate comparison. Although some differences between measurements are evident, it is clearly visible in Table 4 that the thermal conductivity values before and after evacuation were different. The difference is due to the heterogeneity of the lightweight concrete used as the core material. The thermal conductivity values before and after evacuation are more clearly different in the FCP specimen, which had a lower density and a more porous structure than the FCA specimen. In the FCP specimen, it can be seen that thermal conductivity reduced from 16.45 to 27.74% by evacuation, while the FCA specimen showed a thermal conductivity reduction of about 10%. In general, foamed concrete has more pores than lightweight aggregates, with lower porosity affecting the efficiency of evacuation.

A similar trend can be found in the results of the hot plate method. In Table 5, the FCP specimen shows a 26.64% difference in thermal conductivity, while the thermal conductivity of the FCA specimen reduced by 6.64%. Both the Hot Disk and the hot plate methods therefore confirmed that the use of lightweight concrete for the core material can reduce VIP thermal conductivity. Moreover, pure foamed concrete, which is a more porous material, is more effective in enhancing insulation performance via evacuation. In the tables, the thermal conductivity results of the Hot Disk and the hot plate method show a difference. In particular, thermal conductivity is almost double for the FCP specimen; this being due to the measurement capacity of each method, with the hot plate method being able to detect a wider range of thermal conductivity, according to the standard and the manufacturer. In addition, the heterogeneity of the core materials also affects thermal conductivity differences. The Hot Disk results are from specific points, while the results of the hot plate method are of the whole plate specimen; the hot plate method can therefore be considered to be more representative. Nevertheless, both of these standardized measurements show clear differences between before and after evacuation, with the effect of a vacuum being clearly confirmed when lightweight concrete was used as the core material.

### 4.2. Numerical Investigation

**Figure 7:**
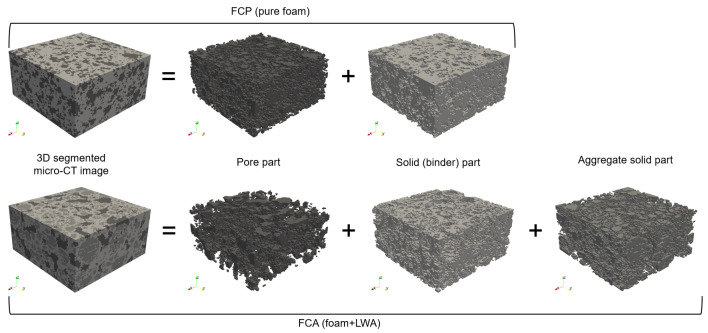
Finite element mesh of the core materials.

For a more detailed analysis of evacuation performance, numerical characterization and simulation were also performed. Thermal simulation was performed using the 3D micro-CT image in Figure 6 by converting it into finite element meshes. Figure 7 shows the finite element meshes of the FCP and FCA specimens. For the FCP specimen, the whole specimen was classified into pore and solid parts, while the FPA specimen was divided into three phases: pore, binder, and aggregate solid.

**Figure 8:**
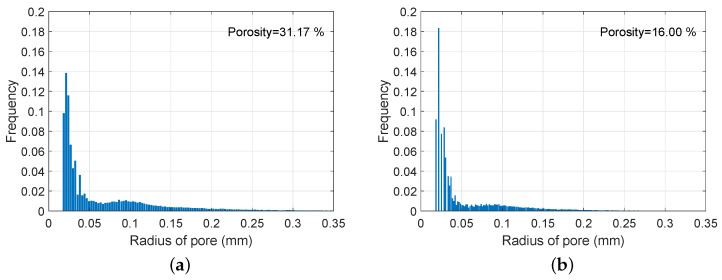
Pore size distribution of the core materials: (**a**) FCP specimen, (**b**) FCA specimen.

The pore structures which strongly affect material properties were investigated using the pore part of each specimen. Based on the used pixel (voxel) resolution, the minimum pore size taken into consideration here was 29.8 μm, with the porosity and pore size distribution being computed using the voxel volume-based information, assuming all the pores were spherical [51,52]. Thus, the pores with the volume less than the image resolution were neglected, and this can affect relatively lower porosity than the actual value due to the limited resolution of the used images. The measured porosity of each specimen was 31.17% (FCP) and 16.00% (FCA). The pore size distributions of the used specimens are presented in Figure 8. In this figure, the total area of the pore size distribution for the FCP specimen is about twice that of the FCA specimen, which is consistent with the porosity trend. In particular, this tendency is more clear for pores larger than 50 μm; the relatively large pores clearly existed in the FCP specimen, while the FCA specimen had a higher proportion of smaller pores (<50 μm). This indicates that pore foamed concrete tended to include larger pores, and that material with lightweight aggregates contained numerous smaller pores since the binder was filled with aggregate particles that include fine pores inside the materials. The measured characteristics confirmed that the general pore structure of foamed concrete can vary, depending on the inclusion of lightweight aggregates.

**Figure 9:**
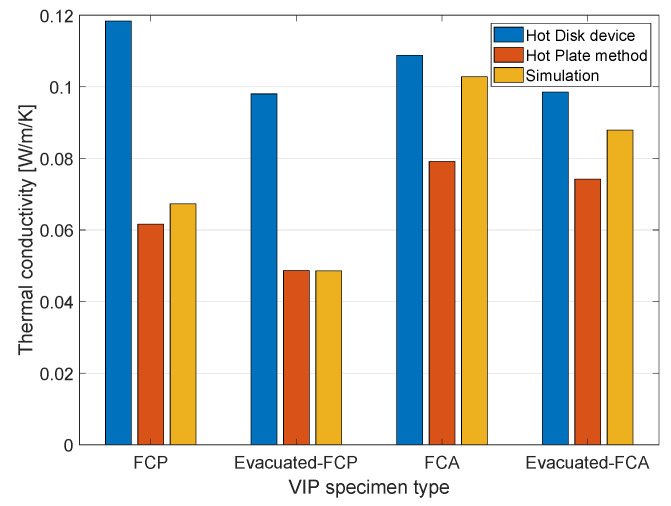
Thermal conductivity of the specimens measured from different approaches.

The numerical analysis for evaluating the thermal conductivity of the specimens was performed using the meshes in Figure 7 and the input parameter introduced in Table 3. The meshes were composed of 400 × 400 × 200 (x×y×z) elements. Figure 9 shows thermal conductivity measured with different approaches, including the experiments in Section 4.1. Both the experiments, the numerically computed thermal conductivity results showed a similar trend; evacuated samples presented lower thermal conductivity than the normal cases, with the difference being clearer in the FCP specimen. The difference was due to the pore properties before and after evacuation. As shown in Table 3, the input thermal conductivity of the pore was lower than that of other solid components and less than half of the lightweight aggregates. In a simulation for the evacuated case, the input value was disregarded, and the effects of the vacuum resulted from the properties of the air. In addition, the porosity affected the degree of thermal conductivity decrease after evacuation. Since the FCA specimen contained less and smaller pores than the FCP specimen, the vacuum effect was less clear than in the specimen with larger and more pores.

**Figure 10:**
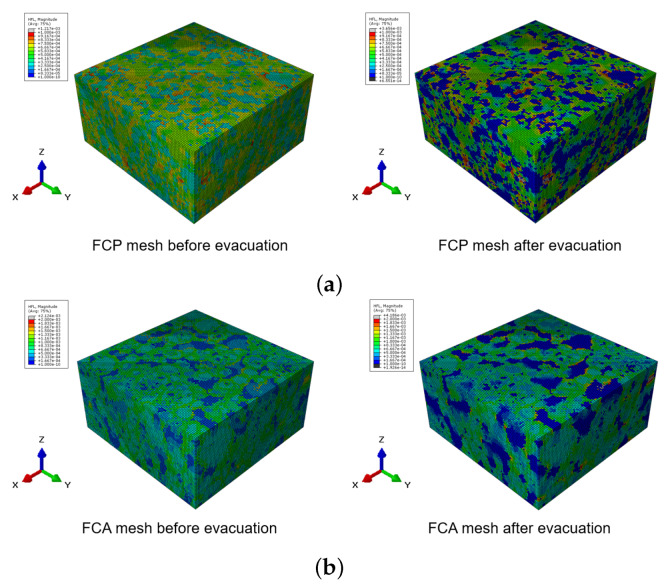
Heat flux contour of the core materials: (**a**) FCP specimen, (**b**) FCA specimen.

**Figure 11:**
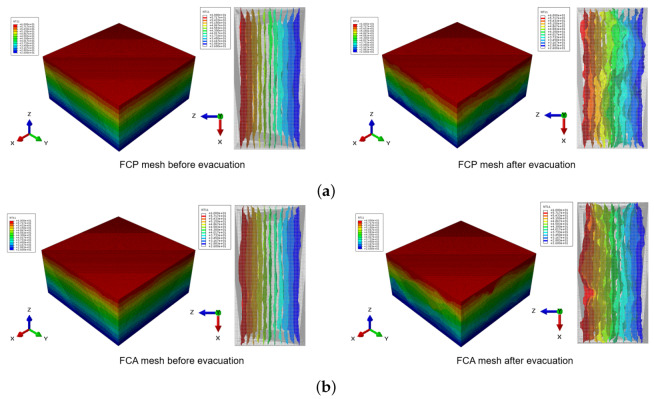
Temperature contour (**left**) and temperature isosurface (**right**) of the core materials: (**a**) FCP specimen, (**b**) FCA specimen.

A detailed investigation of the numerical analysis can be found in Figure 10 and Figure 11. Figure 10 shows the heat flux contour of the specimen before and after evacuation. In each figure, the left image presents the heat flux contour in the normal state, while the image on the right is the heat flux contour of the specimen under vacuum. In these figures, the blue color depicts almost no heat flows in that region. Comparing the images before and after the evacuation, the blue areas were clearer in the vacuum state, since no heat transfer occurred through the pores, with this phenomenon being distinct in the FCP specimen, which had larger porosity and pore size. The effects of evacuation can also be examined in Figure 11. Like the heat flux images, the left and right images of Figure 11 show a temperature contour and its isosurface before and after the vacuum, respectively. In both the FCP and FCA specimens, less fluctuation can be seen in the case of the natural state, while many fluctuations can be found in the vacuum state on the right side, particularly in the vicinity of the pores. Moreover, the difference in fluctuation is more pronounced in the case of the FCP specimen, which contained more and larger pores.

The obtained results confirm that both pure foamed and foamed concrete incorporating lightweight aggregates show a clear difference before and after evacuation. Moreover, it is clear that lightweight concrete of low density, with/without lightweight aggregates, can be effectively used as a VIP core material since it contains numerous pores and has sufficient strength. In addition, the pore structures within the core materials strongly affect the efficiency of evacuation, with a material with large porosity as well as pore size able to be beneficial for achieving better vacuum effects.

## 5. Conclusions

This study aimed to investigate the applicability of lightweight foamed concrete with a low density (270 kg/m${}^{3}$ and 386 kg/m${}^{3}$) as a core material for VIPs. For this purpose, lightweight concrete specimens, pure foamed concrete (FCP) and foamed concrete with lightweight aggregates (FCA), were designed and prepared. The thermal conductivity of the produced specimens was measured both experimentally and numerically, as a parameter for evaluating the effects of evacuation. For a more accurate analysis, two types of standard experimental devices were used and compared. The experimental results were utilized in numerical analysis as input parameters. Micro-CT was also used to visualize and characterize the pore structure of the specimens, with the numerical simulation being performed using the meshes obtained from the CT images.

The concluding remarks of this study are summarized below, as follows:

- Different experimental and numerical approaches show similar trends in the effects of evacuation on lightweight concrete. In both FCP and FCA specimens, regardless of small differences between methods, the FCP specimen showed a lower thermal conductivity than the FCA specimen, with the thermal conductivity clearly decreasing in the vacuum state for both specimens. It is thus confirmed that foamed concrete with a density below 390 kg/m${}^{3}$, with or without aggregates, can be effectively used as a core VIP material.
- The evacuation of lightweight concrete specimens shows a clear effect on insulation, up to a maximum of about 28%. In particular, specimens with less density and more pores shows a clear difference before and after evacuation.
- Pore characteristics, such as porosity and pore size distribution, affect the efficiency of a vacuum. Comparing the cases used in this study, a material with larger porosity and pore size is beneficial in achieving better evacuation and insulation performances.
- Pure foamed concrete with a lower density shows better vacuum efficiency for use as a core VIP material. However, it tends to break during preparation and measurements because of the high evacuation pressure needed for VIP production. To enhance vacuum efficiency, the use of specimens with lower densities but higher strengths is recommended; this can be achieved by using lightweight aggregates or other binders.

In addition, further investigation of the relationship between material characteristics (e.g., pore and solid structures) and evacuation effects needs to be performed. Moreover, parametric studies using different lightweight concretes could be conducted to figure out the effectiveness of lightweight concretes as core VIP materials.

## Figures and Tables

**Table 1:** Chemical composition and physical properties of the used cement and fly ash [wt.%].

Material	CaO	SiO${}_{2}$	Al${}_{2}$O${}_{3}$	Fe${}_{2}$O${}_{3}$	MgO	Na${}_{2}$O	K${}_{2}$O	SO${}_{3}$	Density [kg/m${}^{3}$]	Surface Area [m${}^{2}$/kg] (Blaine)
CEM I 52.5 R	65.9	20.5	3.2	4.8	1.4	0.1	0.4	2.7	3150	386
Fly Ash	4.7	47.8	20.9	4.5	1.5	0.7	1.0	0.9	2270	293

**Table 2:** Mix compositions of foamed concrete.

Mix	Cement (kg/m${}^{3}$)	Fly Ash (kg/m${}^{3}$)	Liaver${}^{®}$ (kg/m${}^{3}$)	w/b	Super Plasticizer (kg/m${}^{3}$)	Stabilizer (kg/m${}^{3}$)	Foam:Paste (vol.)
FCP (pure foam)	140	60	-	0.4	3.0	0.75	4:1
FCA (foam + LWA)	210	90	50	0.4	1.8	0.90	3:1

**Table 3:** The input parameters for the heat transfer analysis.

Specimen	Material	Thermal Conductivity [W/m/K]	Density [kg/m${}^{3}$]	Specific Heat [J/kg/K]
FCP (pure foam)	Matrix	0.205	519	1216
FCA (foam + LWA)	Matrix	0.247	614	1075
	Aggregates	0.075	350	1150
-	Air (pore)	0.035	1.225	1005

(Note: the thermal conductivity of air is $\lambda_{tot}$, where $\lambda_{rad}$ is set to be 0.01 [W/m/K]).

**Table 4:** Thermal conductivity from the Hot Disk device.

Material	Oven-Dry Density (kg/m${}^{3}$)	Point	Thermal Conductivity (W/m/K)		Difference (%)
			Evacuated	Air-Entrained	
**FCP (pure foam)**	270	1	0.1086	0.1288	20.60%
		2	0.0936	0.1090	16.45%
		3	0.0918	0.1173	27.74%
**FCA (foam + LWA)**	386	1	0.0972	0.1073	10.35%
		2	0.1012	0.1100	8.70%
		3	0.0972	0.1091	10.90%

**Table 5:** Thermal conductivity from Hot Plate method and compressive strength.

Material	Oven-Dry Density (kg/m${}^{3}$)	Thermal Conductivity (W/m/K)		Difference (%)	Compressive Strength (MPa)
		Evacuated	Air-Entrained		
**FCP (pure foam)**	270	0.04868	0.06165	26.64%	1.3
**FCA (foam + LWA)**	386	0.07422	0.07915	6.64%	2.4
